# Recent Studies and Applications of Hydrogel-Based Biosensors in Food Safety

**DOI:** 10.3390/foods12244405

**Published:** 2023-12-07

**Authors:** Yuzhen Li, Hongfa Zhang, Yan Qi, Chunping You

**Affiliations:** 1 State Key Laboratory of Dairy Biotechnology, Shanghai Engineering Research Center of Dairy Biotechnology, Dairy Research Institute, Bright Dairy & Food Co., Ltd., Shanghai 200436, China; liyzlyz@163.com (Y.L.); zhanghongfa1977@sina.com (H.Z.); adele0422@126.com (Y.Q.); 2 School of Physical Science and Technology, Shanghai Key Laboratory of High-Resolution Electron Microscopy, ShanghaiTech University, Shanghai 201210, China

**Keywords:** food safety, hydrogel, biosensor, food contaminants, monitoring

## Abstract

Food safety has increasingly become a human health issue that concerns all countries in the world. Some substances in food that can pose a significant threat to human health include, but are not limited to, pesticides, biotoxins, antibiotics, pathogenic bacteria, food quality indicators, heavy metals, and illegal additives. The traditional methods of food contaminant detection have practical limitations or analytical defects, restricting their on-site application. Hydrogels with the merits of a large surface area, highly porous structure, good shape-adaptability, excellent biocompatibility, and mechanical stability have been widely studied in the field of food safety sensing. The classification, response mechanism, and recent application of hydrogel-based biosensors in food safety are reviewed in this paper. Furthermore, the challenges and future trends of hydrogel biosensors are also discussed.

## 1. Introduction

Food safety affects everyone around the world and remains a public health issue of global concern [1,2]. During the stages of cultivation, processing, storage, or circulation, food and agricultural products may be contaminated with various chemical or biological pollutants, such as pesticide residues, veterinary drugs, heavy metal ions, pathogens, and biotoxins [3,4]. These hazardous substances can easily be ingested by the human body through contaminated food or water, causing harm to human health. The World Health Organization estimated that ~420,000 people die from foodborne diseases worldwide each year [5]. The U.S. Department of Agriculture estimated the economic cost of foodborne illness at up to USD 83 billion annually and is rising [6]. So, it is imperative to develop reliable, sensitive, and accurate detection methods to timely monitor food contaminants so as to ensure food safety.

Currently, gold standard methods, e.g., high-performance liquid chromatography (HPLC), gas chromatography (GC), and mass spectrometry (MS), have been widely used in the high-precision quantification of food contaminants [7,8,9]. Despite powerful trace analysis capabilities, the expensive instrumentation, complex operating processes, tedious pretreatment, and lack of trained professionals severely restrict their application in the field of rapid detection, especially in real-time monitoring in emergency situations.

In order to overcome the drawbacks of the above traditional detection technologies, food sensors based on various nanomaterials and biomolecules have been continuously constructed, such as fluorescent, colorimetric, electrochemical, and surface-enhanced Raman scattering (SERS, a non-destructive spectral detection method) sensors, which can quickly and easily detect food contaminants with inexpensive equipment [10,11,12,13]. With strong specificity, high selectivity and sensitivity, convenience, and fast operation, the biosensor detection technology that can realize online addition has been widely used in food safety, such as for detecting food freshness, pathogenic bacteria, pesticide residues, antibiotic residues, heavy metals, and so forth [14]. Recently, due to their highly porous structure, good shape-adaptability, mechanical stability, and high biocompatibility, hydrogels have become a promising biomaterial for designing food safety sensors [15,16].

Hydrogels with highly water-soluble three-dimensional network structures crosslinked by chemical or physical bonds of hydrophilic polymers can be divided into natural hydrogels and synthetic hydrogels [17]. Due to their ability to absorb large amounts of water and swell without being dissolved, structurally stable hydrogels can provide sufficient permeability for the target analytes and a biocompatible microenvironment for enzymes, which is beneficial for improving the sensitivity and stability of the sensors [18]. The porous network structure with the molecular sieve effect allows small targets to spread freely and quickly but keeps large food disturbers out of the surface, which is conducive to improving the anti-interference capability of sensors. Various functional ligands are easily integrated into hydrogels to achieve additional molecular recognition and stimulus–response, which makes it easy to realize the high-sensitivity detection of the sensor. With their soft and flexible properties and high load capacity, hydrogels can be combined with smart devices to build portable inspection platforms. All of these strengths have contributed to the increasing use of hydrogels in food safety sensing applications [3,14]. The biosensor is an independent integrated device that can provide specific quantitative or semi-quantitative analytical results via the direct spatial contact between biometric elements (enzymes, nucleic acids, antibodies, aptamers, etc.) and transducing elements (electrochemical, optical, thermometric, ion-sensitive, or piezoelectric forms) [19,20]. Combining hydrogel with biometric elements, rapid, sensitive, and portable target-responsive hydrogel-based biosensors have been fabricated to realize target recognition and signal amplification, which can meet the needs of real-time quantitative detection [15,21,22]. Although the above advantages exist, the disadvantages of high price and stability also restrict the application of hydrogels in biosensors to a certain extent.

By recording the transformation of detectable optical, electrical, chemical, or biological signals, hydrogel-based biosensors show great potential in the field of food safety detection. Given the rapid development of the field, it is meaningful to review the latest advances in hydrogel-based biosensors for food safety detection. Although there are existing review articles with similar themes [3,10,16,17], the latest applications of hydrogel-based biosensors in food safety detection have not been comprehensively reviewed. This review discusses, compares, and summarizes them. Challenges and future trends in this field are analyzed and discussed.

## 2. Classification of Hydrogels for Sensors

Hydrogels can be classified in many different ways [23]. In terms of the sources, they can be classified into natural and synthetic types. Natural hydrogels, such as chitosan, hyaluronic acid, cellulose, and so on, are used for sensing due to not only their abundant functional groups and unique physiochemical properties but also their good biocompatibility, environmental responsiveness, biodegradability, and swelling behavior [24,25,26,27,28,29,30]. Synthetic hydrogels, including polyethylene glycol (PEG), polyacrylamide (PAM), polyvinyl alcohol (PVA), etc., are suitable for sensors because of their ideal mechanical strength, flexibility, controllable molecular structure, and selective chemical reactivity. Compared to natural hydrogels, synthetic hydrogels have better structural strength due to their stronger covalent bonds [31,32,33].

Hydrogels can be either non-stimulus-responsive or stimulus-responsive on the basis of their responsiveness to external stimuli, and the detection principles of these two types of hydrogels are also completely different. Most non-stimulus-responsive hydrogels are simply used in sensing as solid-phase support substrates for encapsulating or immobilizing nanomaterials, signal probes, or cells, thereby interacting with the target to achieve unique signal transduction. Stimulation-responsive hydrogels, also called smart hydrogels, can sense small changes or stimuli in the external environment (e.g., temperature, pH, light, electricity, and pressure), resulting in changes in the corresponding physical structure or chemical properties, or even mutations, and are mainly used as smart food packaging materials, nutrition delivery systems, and detection sensors in the food field [34,35,36,37]. The photonic, electronic, magnetic, chemical, and mechanical properties of hydrogels can be improved by incorporating nanomaterials into hydrogels [38]. According to the response to different external stimuli, stimulation-responsive hydrogels can be divided into temperature-responsive hydrogels, light-responsive hydrogels, pH-responsive hydrogels, salt-responsive hydrogels, mechano-responsive hydrogels, and multiple external environment-responsive hydrogels.

In recent years, in order to meet the actual needs of different research fields such as food, biology, chemistry, and medicine, various smart hydrogels with specific directional response functions have been rapidly developed [39]. With the development of hydrogel engineering and deoxyribonucleic acid (DNA) self-assembly technology, DNA hydrogels have been studied extensively in the past decades [40]. As a smart hydrogel, DNA hydrogels not only maintain the 3D web-like structures of conventional hydrogels but also integrate the superior properties of DNA, such as programmability, high stability, and precise molecular recognition, which are particularly suitable for biosensors [3]. Specific functions can be obtained by accurately designing the sequence of DNA.

## 3. Recent Application in Food Safety

Over the years, hydrogel-based biosensors have been successfully applied to food safety analysis, such as detecting antibiotic and pesticide residues, heavy metal ions, microbial toxins, pathogenic bacteria, illegal additives, food allergens, and food freshness. Some recent and typical examples are listed in Table 1, including targets, hydrogels used and their functions, real samples, and so on.

### 3.1. Biotoxins

Biotoxins, a kind of toxic substance, are produced through various organisms and widely exist in various foods. Biotoxins can cause acute and subacute diseases and pose a great threat to human health [41]. Biotoxins have become important factors affecting food safety and have caused various food poisoning incidents. It is very important and meaningful to develop accurate, rapid, and convenient detection methods for all kinds of biotoxins in food substrates. Biosensors can detect microorganisms or toxins in food in real time, which greatly ensures the safety of food.

**Figure 1:**
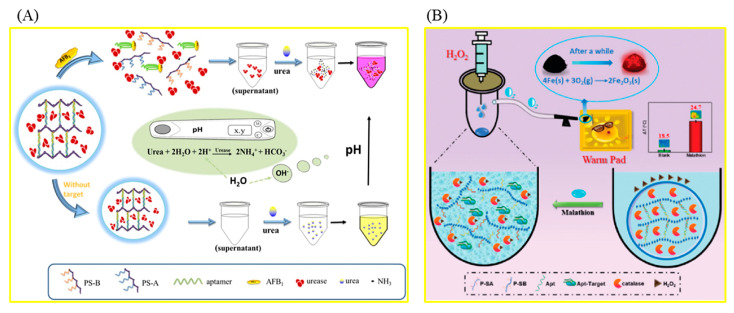
Schematic illustration of a biosensor based on DNA/aptamer hydrogel. (**A**) The portable detection of AFB1 using a pH meter. Reproduced from [45], with permission from Elsevier, 2018. (**B**) The detection of malathion using a thermometer. Reproduced from [61], with permission from RSC, 2021.

DNA/aptamer hydrogels with a web-like structure are often used in the detection of biotoxins, which consist of two polyacrylamide-DNA chains and an aptamer. The specific binding of the target to its aptamer results in a conformational change of the aptamer, inducing the collapse of DNA hydrogel, thereby releasing the coated enzyme molecules or nanoparticles in the cavities of DNA, and finally achieving the homogeneity detection of the target (Figure 1).

Aflatoxin B1 (AFB_1_), classified as a Group 1 carcinogen by the International Agency for Research on Cancer (IARC), is one of the most toxic aflatoxins and can contaminate a wide range of agricultural products, including animal feed, corn, grains, and so on [42,43]. Recently, portable quantitative detection methods for AFB_1_ have been extensively studied. By combining DNA hydrogel and a microfluidic chip, a portable biosensor was constructed to detect AFB_1_ [44]. DNA hydrogel was constructed with the AFB1 aptamer and its two complementary polyacrylamide-DNA strands, and platinum nanoparticles (PtNPs) were loaded into its cavity. The hydrogel can be used together with preloaded PtNPs as a color indicator to detect AFB_1_. After the introduction of a sample solution containing AFB_1_, the AFB_1_ aptamer binds to AFB_1_, resulting in the destruction of hydrogel, the release of PtNPs, and the color of the supernatant changing from colorless to red. PtNPs can catalyze the decomposition of H_2_O_2_ to produce O_2_, which drives the red ink in the chip through the pressure of the O_2_ gas. The concentration of AFB_1_ in the sample can be calculated by measuring the distance that the red ink moves. AFB_1_ in beer was detected with this method, and the limit of detection (LOD) was 1.77 nmol/L. Combining the advantages of the target-responsive aptamer-cross-linked hydrogel and a pH testing system, a simple device for the sensitive determination of AFB_1_ was constructed successfully (Figure 1A) [45]. After adding the sample solution containing AFB_1_, AFB_1_ binds with its aptamer, causing the DNA hydrogel to collapse and release the urease into the solution. The released urease catalyzes urea hydrolysis, resulting in an increase in the pH value of the solution. Portable detection of AFB_1_ can be realized by measuring the pH value of the solution with a pH meter. The detection limit of AFB_1_ using this method was 0.1 µmol/L.

Ochratoxin A (OTA), classified as a Group 2 carcinogen by the International Agency for Research on Cancer (IARC), is a secondary metabolite of a variety of Aspergillus and Penicillium fungi. It is carcinogenic and teratogenic, impairs the immune system, damages the liver and kidney, and leads to neurotoxicity. OTA has been detected in many kinds of food and products. It can damage the liver, kidneys, and immune system, leading to cancer, teratogenicity, and neurotoxicity. Liu et al. [46] successfully detected OTA in beer with a low LOD of 1.27 nmol/L by using DNA hydrogel-loaded gold nanoparticles (AuNPs) for portable and visual quantitative analysis through the volumetric bar-chart chip (V-chip). Using the competition of aptamers, complementary sequences, and targets, a self-assembled fluorescent DNA hydrogel aptamer sensor based on rolling circle amplification (RCA) products was developed for the detection of OTA in beer [47]. T-2 toxin can cause feed refusal [48], reproductive dysfunction, growth retardation, and other diseases, threatening the health of humans and animals. A biosensor based on etched gold nanorods (AuNRs) and DNA hydrogels with T-2 toxin aptamers as crosslinkers was developed to detect the T-2 toxin via a UV signal [49]. The binding of T-2 toxin to the aptamer causes the hydrogel to rupture and release the embedded horseradish peroxidase, which catalyzes the oxidation of KI via H_2_O_2_ to produce I_2_. After being etched using I_2_, the ultraviolet spectrum of AuNRs was blue-shifted. The detection of T-2 toxins can be achieved by using the degree of blue shift proportional to their content.

In addition, a heat-reversible injectable hydrogel formed from Pyrene-based amphiphile could be used to selectively detect Cholera Toxin (CT) via a color-changing response [50]. A portable solar-powered photoelectrochemical biosensor without a light source or workstation has been developed for the detection of OTA in corn juice [51]. The biosensor is made on a small indium tin oxide electrode, which is divided into a detection module and a reference module. Each module consists of a photoelectric sensing area fabricated with the 3D hydrogel of Co and N co-doped TiO_2_ graphene and a visual area constructed with the electrochromic material Prussian Blue. The change in target concentration can lead to a change in the electron migration amount, which causes a color change in the visual area. The chromaticity ratio of the visual area on these two modules of the biosensor has been successfully applied to obtain the concentration of OTA.

### 3.2. Pesticide Residues

Pesticide residue refers to the general term for trace pesticide protoplasms, toxic metabolites, degradants, and impurities in organisms, agricultural products, soil, water, and the atmosphere that are not completely decomposed after pesticide use for a period of time [52]. Pesticide residue is an important factor affecting the sustainable development of the food industry and people’s physical and mental health. If the pesticide residues on fruits and vegetables are seriously excessive, the human body is also very prone to poisoning after ingestion, and life safety is greatly threatened. Sensitive and accurate detection of pesticide residue is an important means to ensure food quality and safety. In recent years, some biosensors based on hydrogels have been successfully applied to the rapid determination of pesticide residues. With the help of smartphones, on-site real-time detections are realized.

**Figure 2:**
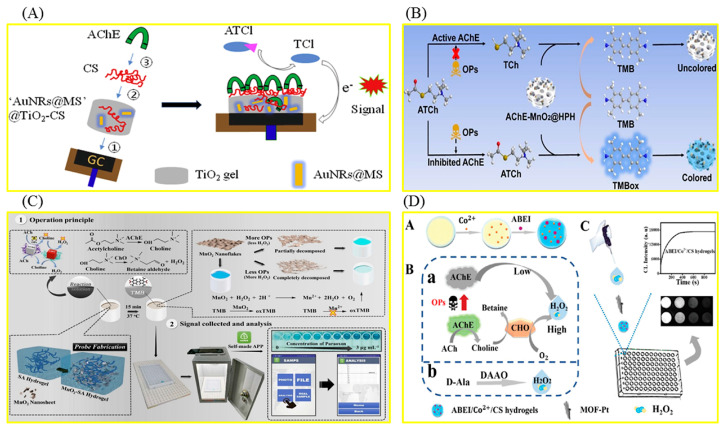
Hydrogel biosensor based on inhibition of AChE via OPPs. (**A**) Fabrication of an AuNRs@MS nanoparticle−doped AChE biosensor and its working mechanism for ATCl. Reproduced from [53], with permission from Elsevier, 2019. (**B**) Sensing mechanism for pesticides using AChE−MnO_2_@HPH. Reproduced from [59], with permission from Elsevier, 2022. (**C**) Schematic illustration of the target−responsive hydrogel smartphone sensor for accurate on−site monitoring of OPPs. Reproduced from [57], with permission from Elsevier, 2021. (**D**) Schematic diagram of detection of OPs and D−AAs Based on MOF−Pt−enhanced long−lasting CL of ABEI/Co^2+^/CS hydrogels. Reproduced from [56], with permission from RSC, 2020.

In order to quickly detect organophosphorus pesticides (OPPs), an important kind of pesticide widely used in agriculture, hydrogel-based biosensors based on the inhibition effect of OPPs on acetylcholinesterase (AChE) are frequently used [53,54,55,56,57,58,59]. The relevant preparation steps and working mechanisms are shown in Figure 2. A sensitive and stable AChE electrochemical biosensor based on this hydrogel for the detection of OPPs was prepared, which can detect dichlovos and fenthion rapidly and accurately. A hierarchical porous hydrogel, AChE-MnO_2_@HPH, was prepared by combining the oxidase-mimetic nanozyme MnO_2_ with the natural enzyme AChE (Figure 2B) [59]. The portable biosensor developed based on the hydrogel can realize the equipment-free, highly sensitive, and in-field detection of fenthion, a model of OPPs. MnO_2_ can catalyze the oxidation of colorless 3,3′,5,5′-tetramethylbenzidine (TMB) to blue TMBox. AChE can hydrolyze the substrate acetylthiocholine chloride (ATCh) to produce TCh with reductive properties, which can inhibit the TMB chromogenic reaction catalyzed via MnO_2_. Fenthion can irreversibly inhibit the activity of AChE, resulting in the restoration of the TMB chromogenic reaction. The color information of the chromogenic reaction was recognized with the self-made smartphone. The biosensor shows excellent stability and can maintain good response performance after 30 days of storage. Polydopamine (PDA)-coated AuPt hydrogels were successfully used to construct an AChE-based biosensor for the detection of paraoxon-ethyl (a model of OPPs) [55]. In order to achieve high throughput screening of pesticides, an array sensor based on N-(4-Aminobutyl)-N-ethylisoluminol/Co^2+^/chitosan (ABEI/Co^2+^/CS) hydrogel was successfully constructed, which, combined with chemiluminescence imaging analysis, can simultaneously identify 17 kinds of OPPs (Figure 2D) [56]. In addition, in order to achieve highly sensitive fluorescence detection of OPPs, a portable hydrogel biosensor was constructed based on tyrosinase, which can regulate the fluorescence intensity of poly-thymine30 DNA-templated copper nanoparticles (PolyT30-CuNPs) [60]. Tyrosinase can quench the fluorescence of PolyT30-CuNPs. After the introduction of OPPs, the fluorescence of PolyT30-CuNPs was recovered due to tyrosinase inhibition. The sensing platform enables the visual and portable detection of OPPs in complex practical samples. DNA hydrogels were also used to detect OPPs. A DNA hydrogel-mediated portable biosensor was designed based on the self-heating reaction (Figure 1B) [61]. Malathion (a model of OPPs) can bind to aptamers in DNA hydrogels to disrupt 3D cross-linked network structures. The released catalase can catalyze the conversion of H_2_O_2_ into oxygen molecules, raising the temperature of the system, which can be measured directly with a thermometer. The biosensor can also be used to detect different pesticides by changing the aptamer sequence in the DNA hydrogel. With the help of a smartphone, an all-in-one test strip integrating nanozymes, bioenzyme assembly, and chromogen realized portable and high-sensitivity detection of paraoxon [62]. The stability of the strip was improved with alginate hydrogel as a carrier. The application of this method to rice, farmland soil, river water, and other real samples proves that it is expected to be an ideal tool for on-site detection of pesticide residues.

Oxalate, a common pesticide adjuvant, can cause damage to human kidneys even in trace residues, but its accurate on-site detection is still a difficult problem. In 2020, a “lab in a tube” platform combining a portable hydrogel kit embedded with MnO_2_ nanosheers with a smartphone was successfully constructed for on-site and accurate monitoring of oxalates [63]. Oxalate can break down MnO_2_ nanosheets, reducing the activity of the nanase to catalyze TMB, resulting in a color reaction in the hydrogel kit. ImageJ software was used to convert the image information collected using the smartphone into hue intensity for the quantitative detection of oxalate. The biosensor enables sensitive, high-throughput analysis of oxalates, screening 12 samples within 10 min.

### 3.3. Antibiotic Residues

Over the years, countries and international organizations around the world have reached a consensus on the many problems caused by antibiotic residues, such as resistance, liver and kidney toxicity, human and animal health, food safety, and the environment [64,65]. Policies and monitoring measures to regulate the rational use of antibiotics have improved, but the abuse of antibiotics still exists. The residual antibiotics are enriched through the food chain and transferred to humans, resulting in a series of toxic side effects that pose potential threats to food safety and human health. Therefore, it is eagerly needed to provide a simple and effective method to detect antibiotic concentrations for early warning.

In 2016, a fluorescent biosensor based on graphene oxide (GO) hydrogel was developed to sensitively detect oxytetracycline (OTC) in water using aptamer and adenosine as the co-crosslinking agents [66]. When the aptamer binds to OTC, the target–aptamer complex is formed, resulting in a significant weakening of the binding between fluorescein imide (FAM) and GO. The increased distance between FAM and GO prevents the transfer of fluorescence resonance energy, resulting in fluorescence.

By trapping plasma core–shell nanoparticles with DNA hydrogel networks, a simple and highly sensitive SERS sensor was developed for the detection of kanamycin—a typical antimicrobial agent [67]. A DNA hydrogel SERS sensor was developed and successfully used for ultrasensitive detection of streptomycin residues in milk and honey samples [68]. An AuNRs array was used to detect the Raman reporter 4-mercaptobenzene, which is released by the triggering of DNAzyme in the hydrogel.

In addition, in order to efficiently adsorb and sensitively detect tetracycline (TC), a non-toxic fluorescent molecularly imprinted hydrogel (FMIH) was constructed based on carbon dots and wood-derived cellulose nanocrystals [69]. The introduction of specific molecular recognition sites realized the sensitive detection and efficient adsorption of TC. The method also provides a new way to simultaneously achieve low-cost, green detection, and the removal of antibiotic residues.

### 3.4. Pathogenic Bacteria

This microbial contamination of food remains a worldwide problem. Plate counting and quantitative real-time polymerase chain reaction (PCR) methods for bacterial detection are accurate but time-consuming or demanding for operators, so rapid, non-invasive, and accurate monitoring of pathogenic bacteria in food has always been the goal of researchers [70]. According to the characteristics of bacteria, hydrogels with 3D porous nanostructures can select different types of hydrogels to improve the biological activity and stability of biological components (enzymes, antibodies, antigens, etc.). Bacteria interact with hydrogels mainly through electrostatic interaction, hydrophobic bonds, and hydrogen bonds, but these interactions are not targeted or specific. Therefore, enzymes, antibodies, and polysaccharides are often added to the hydrogel matrix to enhance its selectivity and sensitivity [71].

PH-responsive hydrogel-based biosensors are also commonly used to quantify *Escherichia coli* (*E. coli*) [72,73]. Dihydrolipoic acid-capped Ag nanoclusters doped with agarose hydrogels were used as a pH “OFF-ON” signal switch to detect the change in pH value in the process of bacterial growth and metabolism with a range from 8.0 to 4.0 [72]. With the decrease in pH, the fluorescence of the hydrogels was weakened and completely quenched after pH 5. Electrospun poly acrylic acid (PAA)/PVA hydrogel nanofibers were integrated into the Light Addressable Potentiometric Sensor (LAPS) by Shaibani, P. M. et al. to make a biosensor that can detect *E. coli* quickly and effectively [73]. The biosensor with a super-Nernstian response in water pH has a theoretical LOD of 20 CFU/mL for *E. coli*. The nanofiber-LAPS was also used to fabricate a portable biosensor to rapidly detect *E. coli* in orange juice with an LOD of 100 CFU/mL [74].

Based on the different enzymes produced by various bacterial strains and the selectivity of enzyme cleavage reactions of dye fluorescent substrates bound to hydrogels, a chitosan hydrogel-based sensing platform for the detection of *E. coli* O157: H7 was developed, which can effectively distinguish pathogenic from non-virulent *E. coli* [75]. A sensitive proportional photoelectrochemistry biosensor was prepared for the detection of E. coli using three-dimensional graphene hydrogel-loaded carbon quantum dots (C-dots/3DGH) modified by *E. coli* aptamer and graphene-like carbon nitride (g-C_3_N_4_) [76]. The concentration of *E. coli* can be quantified sensitively by the ratio of the cathodic current generated by C-dots/3DGH to the anodic current produced by g-C_3_N_4_ without interference from external factors. This ratiometric PEC biosensor without additional instruments showed good sensitivity (0.66 CFU/mL). A nanoporous PEG hydrogel controlled release was developed by Lin et al. for digital quantification of single pathogenic bacteria and nucleic acid rapidly and precisely in untreated food samples [77]. This multifunctional hydrogel with adsorption, release, restriction, and separation abilities can directly quantify *E. coli* and Salmonella typhi in fresh fruit and vegetables within 20 min with high accuracy and sensitivity.

### 3.5. Heavy Metals

In recent years, heavy metal pollution has become more and more serious, which has brought great threats to the environment and human health. Therefore, accurate monitoring of heavy metal ions becomes particularly important. As an excellent biological monitoring technology, biosensors have been widely used in the detection of heavy metal ions [78,79,80,81,82].

As early as 2014, hydrogel biosensors were developed for the rapid visual and quantitative detection of trace amounts of lead [78]. The hydrogel was prepared by using Pb^2+^-dependent DNAzyme and its substrate as the cross-linking agent. For quantitative visual detection, gold-platinum core–shell nanoparticles (Au@PtNPs) embedded in the hydrogel were used to read out the volumetric bar-chart chip (V-chip). The DNAzyme in this method is fungible, and if it is replaced by another responsive DNAzyme, the method can be further expanded to include the visual quantitative detection of other targets. In 2020, a portable DNA-based hydrogel capillary sensor for point-of-care detection of Pb^2+^ was proposed based on the network structures of the DNA hydrogel and the capillary action of the capillary tube [82]. Due to the obstruction of the DNA hydrogel film at the end of the capillary, the blank solution cannot flow into the capillary. When the solution contains Pb^2+^, the substrate of the crosslinker Pb^2+^-dependent GR-5 DNAzyme is split, causing the hydrogel film to partially break, allowing the solution to flow into the tube. With the increase in Pb^2+^ concentration, the solution enters the capillary faster. Pb^2+^ in tap water can be quantitatively detected using a visual reading of distance and duration. The biosensor enables portable, sensitive, in situ visual detection of Pb^2+^. This year, a portable and inexpensive distance biosensor based on paper was developed to detect Pb^2+^ in water with the aid of a DNA hydrogel [83]. The concentration of Pb^2+^ can be obtained by measuring the flow distance of water on the pH paper without the use of special instruments.

### 3.6. Food Quality Indication

Food quality inspection plays an important role in the processes of food production, transportation, storage, and sale. Traditional analytical methods for food quality monitoring, such as chromatography and mass spectrometry, are expensive, complicated, and not easy to carry. Therefore, as a fast-food quality detection method, biosensors have the advantages of high sensitivity, low cost, and portability and have been widely used to detect nutrition or harmful substances in food [84]. It can monitor the freshness or spoilage degree of food by detecting the presence and content of certain substances in food, so as to control the safety and quality of food. However, moisture in food can affect the sensitivity of biosensors. Therefore, the application potential of hydrogels in food quality detection and other food fields is gradually emerging. Hydrogels with high hydrophilicity, water retention, and biocompatibility can effectively reduce the interference of food moisture and become ideal materials for the development of biosensors and indicators [85,86,87].

Food spoilage is a complex process that is affected by many factors, such as temperature and humidity. Temperature is one of the key factors affecting the quality of most foods. High temperatures and large-scale temperature changes are easy to cause food deterioration, which is not conducive to food storage. Humidity also has a great impact on food. If the humidity in the package is too low, foods with high water activity, such as meat, fruits, vegetables, and other fresh products, will lose water and become dry, which will absorb water and spoil as the humidity is too high [88]. In addition, quality changes in many foods are accompanied by changes in their pH and oxygen content. Microbial destruction of food produces acidic or alkaline metabolites that can change the pH of the food samples [89]. It is a feasible method to evaluate the degree of food spoilage by detecting temperature, humidity, pH value, and oxygen content [90]. The application of pH response dyes in hydrogel matrix to detect food freshness is the most widely studied. Nanocellulose was prepared from bagasse cellulose fiber and then crosslinked with Zn^2+^ to form a stronger hydrogel matrix, a carrier for a pH response dye [85], which can change color according to the freshness of the chicken sample. The color of the hydrogel changed distinctly depending on the freshness of the chicken, indicating the hydrogel can be used as a real-time food freshness indicator. The cellulose-chitosan matrix hydrogels with carrot anthocyanins were used as colorimetric dye-based pH indicators to monitor the freshness of pasteurized milk [91].

This kind of hydrogel, with its indicating function, can be used for intelligent packaging of food as a novel type of material that integrates the functions of packaging, testing, and recording. It can provide real-time quality information on food to satisfy people’s demand for high-quality and safe food. It can be seen that the application of hydrogels in food quality detection has great potential.

### 3.7. Other Applications

In addition, hydrogel biosensors have been used for the detection of food additives illegal additives, and allergens.

Nitrite ions are widely used as food additives that can improve the texture, color, and storage stability of food; however, they can produce cancer-causing nitrosamines during cooking [92,93]. Because nitrite ions are important indexes of food safety and water quality, the detection of nitrite ions has received more and more attention from researchers. Unlike other expensive and time-consuming chromatography and electrophoresis [94,95], colorimetric nitrite analysis (for example, the Griess assay) is a portable, low-cost, and rapid detection method [96]. In 2018, a biosensor for the rapid detection of nitrite ions was developed using PEG hydrogel-perimposed glass fiber film strips [97]. A Griess reagent, N-(1-naphthyl)ethylenediamine, was covalently fixed to the hydrogels. On the hydrogel sensor, nitrite ions were detected using the colorimetric Griess method.

Illegal additives, such as melamine, Sudan red dye, malachite green, and so on, are banned in food in many countries. Although they are seriously adverse to human health, they are still used in food by criminals due to their interests [98]. Hydrogel-based biosensors have been reported for the sensitive detection of illegal additives. Sun et al. employed metal nanoparticles (MNPs) embedded in poly(ethylene glycol)-diacrylate (PEGDA) hydrogels as SERS substrates for reliable, fast, and pretreatment-free detection of melamine in milk with a LOD of 10 nmol/L [99]. Similar, flexible MNP-based hydrogel SERS substrates were also applied for the sensitive detection of Sudan red on fruit peel and malachite green in tilapia fillets [100,101].

Food allergen detection is very necessary. Many foods, such as eggs, soy, and milk, are reported to contain allergens that can lead to anaphylaxis, affecting about 6~8% of children and 2% of adults [102]. Based on 3D printing technology, the conductive hydrogels have been developed into an “intestinal microvilli” biosensor for the accurate and specific detection of wheat gliadin (a kind of food allergen) [103]. The detection limit was as low as 0.036 ng/mL. The mixing of copper oxide nanoparticles and multi-walled carbon nanotubes increased the conductivity of the hydrogel, resulting in the detection limit of the biosensor reaching 0.036 ng/mL.

Table 2 gives a comparison of several typical hydrogel-based biosensors. It can be seen that DNA hydrogel-based biosensors are the most widely used and show unique advantages.

**Table 1:** Typical recent applications of hydrogel-based biosensors in food safety (2016–2023).

Target	Hydrogel	Function	Method	Detection Range	LOD	Real Sample	Ref.
**Biotoxins**							
AFB_1_	DNA hydrogel	Encapsulate aptamer, controlled release system	pH	0.2–20 µmol/L	0.1 µmol/L	Corn, peanut	[45]
OTA	DNA hydrogel	Encapsulate aptamer	Fluorescence	0.05–100 ng/mL	0.01 ng/mL	Beer	[47]
T-2 toxin	DNA hydrogel	Target-responsive to release HRP	Fluorescence	0.01–10,000 ng/mL	0.87 pg/mL	Coffee, corn, soybean	[49]
CT	Supramolecular hydrogel	Target-responsive	Color-changing	0–5 µmol/L	-	Water	[50]
OTA	3D graphene hydrogel	Supporting nanoparticle	Photoelectrochemical	1–100 ng/mL	0.29 ng/mL	Corn juice	[51]
**Pesticide residues**							
Paraoxon-ethyl	Polydopamin-capped AuPt hydrogel	Immobilize AChE	Electrochemical	0.5–1000 ng/L	0.185 ng/L	Tap water, lake water	[55]
Chlorpyrifos	Chitosan hydrogel	-	Chemiluminescence	0.5–1000 ng/mL	0.21 ng/mL	Pakchoi	[56]
Fenthion	AChE-MnO_2_@HPH	Immobilize AChE	Color information	4–400 ng/mL	0.63 ng/mL	Rice, wheat	[59]
Paraoxon	DNA hydrogel	Encapsulate CuNPs	Fluorescence	0.1–1000 ng/mL	0.0333 ng/mL	Tap water	[60]
Malathion	3D DNA hydrogel	Encapsulate catalase	Thermal	0.0001–10 ng/mL	0.032 pg/mL	-	[61]
Paraoxon	Alginate hydrogel	Carrier	Colorimetric	0.397–79.4 ng/mL	0.115 ng/mL	Six kinds ^b^	[62]
**Antibiotic residues**							
Kanamycin	DNA hydrogel	Encapsulate GCNPs	SERS	1–10^4^ pg/L	2.3 fmol/L	Milk, honey	[66]
Streptomycin	DNA hydrogel	Incorporate DNAzyme, target-responsive	SERS	0.01–150 nmol/L	4.85 pmol/L	Milk, honey	[67]
Tetracycline	Molecularly imprinted hydrogel	Encapsulate carbon dots, absorb tetracycline	Fluorescence	0.2–1.0 µg/L	0.11 µg/L	Tap water, lake water	[68]
**Pathogenic bacteria**							
*E. coli*	PVA/PAA hydrogel	pH-sensitive hydrogel	pH	10^2^–10^6^ CFU/mL	10^2^ CFU/mL	Orange juice	[74]
*E. coli*	3D graphene hydrogel	Encapsulate carbon dots	Photoelectrochemical	2.9–2.9 × 10^6^ CFU/mL	0.66 CFU/mL	Milk	[76]
*Salmonella typhi, E. coli*	Cross-linked PEG hydrogel	Conduct LAMP inside	Fluorescence	1–640 copy/µL	0.4 copy/µL	Fruit, vegetable	[77]
**Heavy metals**							
Pb^2+^	PNBC hydrogel ^a^	Embed Fe_3_O_4_	Fluorescence	10^−3^–10 mmol/L	-	Water	[81]
Pb^2+^	DNA hydrogel	Target-responsive	Distance and time	0.01–50 µmol/L	10 nmol/L	Tap water	[82]
Pb^2+^	DNA hydrogel	Target-responsive	Distance	0–200 nmol/L	0.3 nmol/L	Lake water, Tap water	[83]
**Food quality indication**							
CO_2_	Nanocellulose hydrogel	pH indicator	Colorimetric	0.1–56.5% (*v*/*v*)	/	Chicken breast	[85]
Total mesophilic counts	Cellulose/chitosan	pH indicator	Colorimetric	3–7.65 (log10 CFU/mL)	/	Milk	[91]
Other applications							
Melamine	PEGDA hydrogel micropellet	Encapsulate MNPs	SERS	10^−8^–10^−3^ mol/L	10 nmol/L	Milk	[99]
Wheat gliadin	Conductive hydrogels	Immobilize cell	Electrochemical	0.1–0.8 ng/mL	0.036 ng/ml	Gluten-free flour and cookies	[103]

^a^ Poly(N-isopropylacrylamide-co-benzo-18-crown-6-acrylamide). ^b^ Rice, wheat, rapeseed, farmland soil, Yudai River water, and Yangtze River water.

**Table 2:** Comparison of several typical hydrogel-based biosensors in food safety.

Hydrogel	Target	Function	Method	Ref.
DNA hydrogel	AFB_1_, OTA, Paraoxon, Malathion, T-2 toxin, Kanamycin, Streptomycin, Pb^2+^	Encapsulate aptamer, controlled release system, target-responsive	pH, fluorescence, thermal, SERS, distance and time	[45,47,49,60,61,66,67,82,83]
3D graphene hydrogel	OTA, *E. coli*	Supporting nanoparticles, encapsulate carbon dots	Photoelectrochemical	[51,76]
Chitosan hydrogel	Chlorpyrifos	pH indicator	Chemiluminescence, colorimetric	[56]
Supramolecular hydrogel	CT	Target-responsive	Color-changing	[50]
Molecularly imprinted hydrogel	Tetracycline	Encapsulate carbon dots, absorb tetracycline	Fluorescence	[68]
Cross-linked PEG hydrogel	*Salmonella typhi, E. coli*	Conduct LAMP inside	Fluorescence	[77]

## 4. Conclusions and Future Prospects

Hydrogel-based biosensors have witnessed tremendous development in food safety. Many papers on this area have been published, and there is a growing trend. This review first introduces the classification of hydrogels, briefly describes the characteristics of various hydrogels, and then focuses on the recent application of hydrogel-based biosensors in food safety aspects such as biotoxins, pathogens, pesticide residues, antibiotic residues, heavy metal ions, food additives, allergens, and food quality indicators. The flexible porous structure, large load capacity, excellent biocompatibility, and good shape adaptability of hydrogel not only make the biosensor realize fast, portable, and sensitive detection but also improve the anti-interference ability. Hydrogel-based biosensors can even enable the detection of complex substrates without preprocessing.

However, despite great promises and advancements in hydrogel-based biosensors for food safety, there are still several challenges with great potential that need to be addressed, especially in practical applications. These challenges provide ample scope for future research and development, as the field is evolving very rapidly. Efforts can be made in the following aspects in the future:

1. The development of hydrogel-based sensors is limited by the structure and function of polymers, so new cross-linking methods should be used to design and synthesize polymer networks to achieve precise, targeted regulation of functions;
2. Simulation technology (such as Monte Carlo, molecular dynamics, and multiphysics simulation) can be applied to the structural design and structure–activity relationship interpretation of hydrogel biosensors;
3. Multiple-target detection platforms should be built to meet the needs of multi-channel fast detection;
4. In order to optimize the structure of hydrogels and improve the stability and selectivity of hydrogel biosensors, it is necessary to study the interaction mechanism between hydrogels and food substrates;
5. With the rapid advancement of big data and portable devices, miniaturization and wearable intelligent instruments should be developed to meet the needs of on-site real-time monitoring and rapid government regulation;
6. The continuous dynamic real-time monitoring device should be developed by making full use of the structural characteristics of hydrogels;
7. In order to reduce the cost of hydrogel biosensors, new multifunctional hydrogels should be developed;
8. At present, ethics and privacy are not involved in biosensors in food safety, but in promoting the development of biosensor technology, they will also be included in our future considerations to ensure the proper and safe use of technology.

In conclusion, we believe that through multidisciplinary cooperation, more low-cost, responsive, ultra-sensitive, and environmentally friendly hydrogel-based biosensors are expected to make a greater contribution to food safety.

## Data Availability

Data are contained within the article.
